# The magnitude of second-trimester induced abortion and associated factors in Ethiopia: a systematic review and meta-analysis

**DOI:** 10.3389/fgwh.2025.1535329

**Published:** 2025-07-01

**Authors:** Maru Mekie, Setegn Muche Fenta, Wassie Yazie Ferede, Enyew Dagnew Yehuala, Eyaya Habtie Dagnaw, Alemu Degu Ayele, Temesgen Dessie Mengistu, Belaynew Alemye Mengistie, Selamawit Girma Tadesse, Dagne Addisu

**Affiliations:** ^1^ Department of Midwifery, College of Health Sciences, Debre Tabor University, Debre Tabor, Ethiopia; ^2^ Department of Statistics, College of Natural and Computational Sciences, Debre Tabor University, Debre Tabor, Ethiopia; ^3^ Maternal and Child Health Officer, Debre Tabor Comprehensive Specialized Hospital, Debre Tabor, Ethiopia

**Keywords:** induced abortion, associated factors, second trimester, review - systematic, Ethiopia

## Abstract

**Background:**

Even though Ethiopia has a non-restrictive abortion law, abortion complications are one of the top five maternal morbidity and mortality causes in the country. Most women visit health facilities for pregnancy termination at second-trimester which leads to higher abortion-related complications than first-trimester abortion. There is no national evidence regarding the level of second-trimester-induced abortion in Ethiopia. This study aimed to determine the magnitude of second-trimester induced abortion and its determinant factors.

**Methods:**

Online searches using different online bases such as PubMed, HINARI, SCOPUS, Google Scholar, and University digital libraries were conducted to identify candidate studies to be included in this systematic review and meta-analysis. The Newcastle-Ottawa Quality Assessment Scale (NOS) was used to assess the quality of studies to be included in this review. Data extraction and analysis were performed using Microsoft Excel and Stata 17 software respectively. The heterogeneity of studies was assessed using Cochran (Q test) and I^2^ test statistics. We assessed publication bias using a funnel plot and Egger's regression asymmetry test.

**Results:**

Eight studies with a total study population of 3,659 were included in this review. The pooled prevalence of second-trimester induced abortion was 25.96% (95%, CI 14.42%, 37.49%) in Ethiopia. The finding of this systematic review indicated that being single [(OR = 5.20, 95%, CI 3.04, 8.90), *I*^2^ = 0.00%, *p* = 0.69], delay in the diagnosis of pregnancy [(OR = 3.01, 95%, CI 1.23, 7.38), *I*^2^ = 80.74%, *p* = 0.01], no formal/low education level [(OR = 3.54, 95%. CI 1.84, 6.78), *I*^2^ = 69.71, 57.15%, *p* = 0.04], and being rural resident [(OR = 2.16, 95%, CI 1.61, 2.92), *I*^2^ = 0.00%, *p* = 0.53] were factors significantly associated with second trimester induced abortion in Ethiopia.

**Conclusion:**

The prevalence of second-trimester abortion was found to be high in Ethiopia. Being single, delay in the diagnosis of pregnancy, having no formal/low education level, and being rural residents were factors significantly associated with second-trimester induced abortion in Ethiopia. Enhancing the sexual and reproductive health literacy of reproductive-age women as well as access to safe abortion services are relevant measures to be taken to reduce late visits to health institutions for abortion services.

## Background

Ethiopia is one of the low- and middle-income countries (LMICs) with the highest burden of maternal mortality in the world. The maternal mortality ratio (MMR) was reported to be 412/100,000 live births in the country based on the evidence from the Ethiopian Demographic and Health Survey (EDHS) (1). Despite there being a significant decline in induced abortion rates at the global level, there is no significant change in low- and middle-income countries (LMICs) (2).

An estimated 620,300 induced abortions were performed in Ethiopia in 2014. According to the study, the annual abortion rate was 28 per 1,000 women aged 15–49 years with the highest in urban regions (3). The majority of induced abortions result from unintended pregnancies, which could be averted by improving access to and proper utilization of contraceptives (2). According to the United Nations, one in ten women worldwide reported that they are not using any methods of contraceptives despite their interest in stopping or delaying pregnancy (4). The magnitude of unmet need for family planning was reported to be 22% as per the EDHS 2016 report (1).

To promote modern healthcare and reduce maternal and child mortality, the Government of Ethiopia implemented healthcare reforms in 2005 with the introduction of the Health Sector Development Plan. This reform ensures that maternal and child health services are provided free of charge at public facilities. The services offered at no cost include antenatal care, labor and delivery assistance, emergency obstetric care (including cesarean sections), family planning, and health education. The provision of free maternal health services has significantly contributed to lowering maternal and child morbidity and mortality rates by increasing service utilization and eliminating economic barriers to access (5–7).

“The new WHO guideline recognizes that improving access to abortion care is part of establishing an enabling environment for universal health coverage that ensures all segments of the population receive quality care without financial constraints” (8). Evidence indicated that the average maternal mortality ratio was significantly higher among countries with restrictive abortion policies than countries with liberal abortion policies (9).

Ethiopia has relatively liberal abortion regulations, following a revised law enacted in 2005. Termination of pregnancy by a recognized medical institution within the period permitted by the profession is not punishable where: pregnancy as result of rape or incest, when the pregnancy poses a risk to the mother's or child's life, if the fetus has a serious and incurable deformity, or if the pregnant woman is physically or mentally unfit to raise a child due to a deficiency or her age (ages < 18 years). Meeting just one of these conditions is sufficient to access safe abortion services, and women can self-report is enough as evidence to obtain these services (10).

Although there have been improvements in abortion-related morbidity and mortality, complications from abortion remain one of the top five causes of maternal morbidity and mortality in the country. Most women visit health facilities for pregnancy termination in the second trimester which increases abortion-related complications (11). Though there is a decline in abortion-related mortality in Ethiopia, there is still significant abortion-related maternal morbidity and mortality in the country (12).

Second-trimester-induced abortion results in more complications compared to first-trimester-induced abortion (13). Delay in abortion care significantly increases the health risks and economic burden experienced by women. Abortions performed after 12 weeks of gestation pose greater risks of medical complications than abortions performed during the first trimester. The risk of complication is reported to be 0.3 per 100,000 for abortions done before 10 weeks compared to 12.7 per 100,000 for abortions done after 21 weeks and beyond (14). The magnitude of delayed decisions for safe abortion services was reported to be 70.8% in a study done in health facilities in Southwest Ethiopia (15). Adolescents, younger women, women living further from clinics, women who need to travel for abortion, women with lower educational attainment, women facing financial hardship, and unemployed women” are more likely to visit health facilities for abortion services late in pregnancy (8). If it is not possible to avoid unwanted pregnancy that leads to abortion, early health seeking is important to prevent abortion-related complications.

There is a great disparity between the proportion of second-trimester induced abortion among different individual studies in Ethiopia which ranges from 18.20% to 53.36% (16–21). However, to the best of the authors' knowledge, there is limited national evidence regarding the burden of second-trimester abortion and its contributing factors. Having comprehensive evidence regarding the magnitude will be relevant to take management measures. Hence, this review aimed to assess the magnitude and associated factors of second-trimester induced abortion in Ethiopia.

## Methods

### Study design

We conducted a systematic review and meta-analysis using the evidence obtained from observational studies conducted in different parts of Ethiopia. Ethiopia revised its abortion law in 2005, and we aim to evaluate the prevalence of second-trimester induced abortions and its determinant factors after a decade following the revised abortion law.

### Search strategy and study selection

We employed electronic databases and digital libraries to search for studies conducted about second-trimester abortion and its associated factors in Ethiopia. As a result, both published peer-reviewed articles and unpublished/gray literature were incorporated into this study. Only studies published in English were considered for this systematic review and meta-analysis. Online searches were conducted across various platforms, including PubMed, HINARI, SCOPUS, Google Scholar, and university digital libraries to identify potential studies for inclusion in this systematic review and meta-analysis.

**Figure 1:**
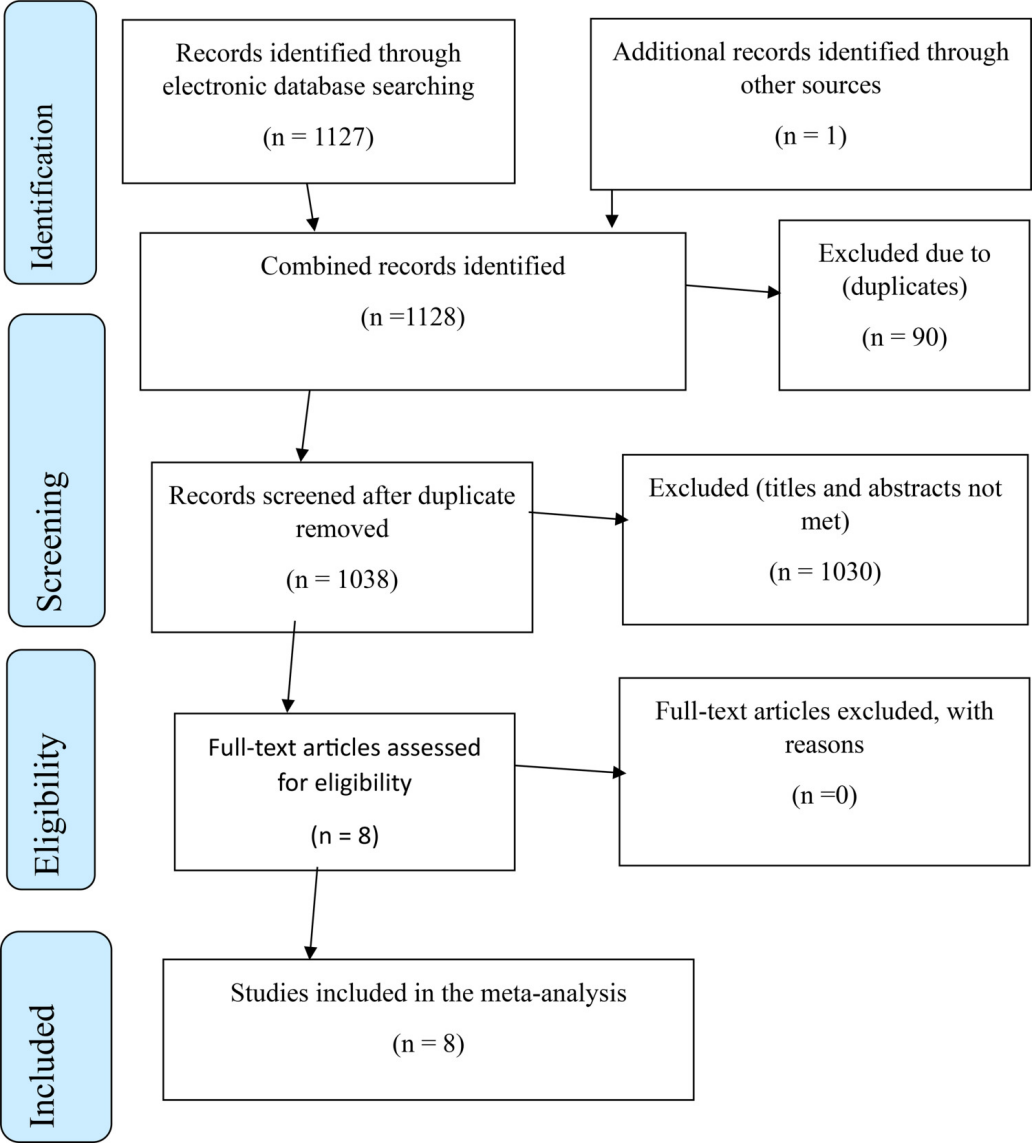
PRISMA flow chart indicating study inclusion and exclusion for systematic review and meta-analysis second trimester induced abortion in Ethiopia.

PICO (P = Population, I = Intervention, C = Comparison, and O = Outcome) strategy was used to formulate search terms to identify candidate studies. in identifying the candidate studies (22). P (Reproductive age women), I (second trimester induced abortion), O (Level of induced second-trimester abortion), C (comparison of the magnitude of second trimester abortion with different characteristics). Epidemiology, prevalence, second trimester of pregnancy, and associated factors were the search terms used in this study (Table 1). A literature search was carried out from December 15, 2023, to January 1, 2024. The details regarding the inclusion and exclusion criteria for studies are outlined according to the Preferred Reporting Items for Systematic Reviews and Meta-Analyses (PRISMA) guidelines (23) (Figure 1).

**Table 1:** Search strategies used for PubMed and related databases to identify candidate studies for the systematic review and meta-analysis.

Databases	Search terms	Number of studies
PubMed/Medline	((“epidemiology"[Subheading] OR “epidemiology"[All Fields] OR “prevalence"[All Fields] OR “prevalence"[MeSH Terms]) AND (“pregnancy trimester, second"[MeSH Terms] OR (“pregnancy"[All Fields] AND “trimester"[All Fields] AND “second"[All Fields]) OR “second pregnancy trimester"[All Fields] OR (“second"[All Fields] AND “trimester"[All Fields]) OR “second trimester"[All Fields]) AND (“abortion, induced"[MeSH Terms] OR (“abortion"[All Fields] AND “induced"[All Fields]) OR “induced abortion"[All Fields] OR “abortion"[All Fields]) AND associated[All Fields] AND factors[All Fields] AND (“ethiopia"[MeSH Terms] OR “ethiopia"[All Fields])) AND (“2014/01/01"[PDat]: “2024/01/01"[PDat])	1030
Other databases		97
Gray literature		1
Total Search		1128
Numbers of candidates for inclusion		8
Excluded with reasons		0
Studies included in the analysis		8

### Inclusion and exclusion criteria

#### Inclusion criteria

Studies conducted about the magnitude of second-trimester induced abortion and or associated factors published in an open access journal in the English language from January 1/2014 to January 1/2024 or unpublished studies conducted from January 2014 to January 2024 were included in this systematic review and meta-analysis.

#### Exclusion criteria

The exclusion criteria included studies that did not meet the inclusion requirements and studies conducted about spontaneous second-trimester abortions since our review focuses on induced second trimester abortion.

### Data extraction

Mekie has created a data extraction form within a Microsoft Excel workbook to gather information on the prevalence of second-trimester induced abortions and their determining factors. The Excel worksheet includes fields for the author's name, publication year, study region, study site, study design, study period, sample size, prevalence, standard error of prevalence, log odds ratio (logor), and standard error of the logor for data extraction purposes.

### Measurement of the outcome and independent variables

#### Second trimester induced abortion

In this review second trimester abortion was defined as termination of pregnancy after 13 completed weeks up to 28 weeks of gestation and terminated either medically or surgical methods (10).

#### Residence

Urban residence was defined as areas with a minimum population of 2,000, while rural residence was classified as areas characterized by sparse populations or those with fewer than 2,000 residents as defined by ministry of Urban development and construction of Ethiopia (24).

#### Delayed diagnosis of pregnancy

This variable was defined as a woman's inability to recognize pregnancy or to identify pregnancy symptoms beyond five weeks that leads to late seeking to safe abortion services (15, 21).

#### Education level

In this review, education level was categorized into two groups: No/Low Education Level and Secondary Education and Above. No/Low Education Level refers to individuals who have received no formal education or have completed only primary education (grades 1–8) or less. In contrast, Secondary Education and Above includes those who have completed secondary education (grades 9–12) or have attended college or higher (15, 21, 25).

### Data quality assurance and data analysis

Studies conducted about the magnitude of second-trimester induced abortion were exported to Endnote 8 to remove duplicates after an independent review of titles and abstracts by two authors, Mekie, and Addisu independently. Disagreements on inclusion and exclusion of studies were resolved through discussion including the third (Ferede) and the fifth (Daganaw) authors. The quality of the included studies was critically assessed using the Newcastle-Ottawa Quality Assessment Scale (NOS) to be included in this systematic review and meta-analysis. Sample representativeness, sample size, no-response rate, ascertainment of risk factors, comparability of subjects in different outcomes, control of confounding factors, assessment of the outcome, and statistical test used were the criteria to critically appraise the quality of included studies. Thus, studies with quality scores of ≥7 were considered as low risk for bias in the NOS quality assessment scale (Table 2).

**Table 2:** Characteristics of the included studies in the systematic review and meta-analysis of the second trimester induced abortion in Ethiopia.

Author	Publication year	Region	Study area	Study design	Sample size	Population of outcome	Prevalence	Response rate	Risk of bias
Abebe et al. (16)	2022	South Ethiopia	Wolayita, Gamo health facilities	Cross-sectional	353	82	23.23	100%	Low risk
Bonen et al. (25)	2014	Oromia	Jimma Health facilities	Cross-sectional	808	100	12.37	100%	Low risk
Mizuna et al. (15)	2020	Oromia	Jimma Health facilities	Cross-sectional	348	——	—–	100.00%	Low risk
Tesfaye et al. (20)	2020	Amhara	Debre Markos health facility	Cross-sectional	262	78	29.77	94.0%	Low risk
Wasihun et al. (21)	2021	Amhara	Amhara health facilities	Case-control	357	———	—–	100%	Low risk
Malate et al.	2015	Amhara	Referral hospitals	Cross-sectional	422	81	19.19	98.6	Low risk
Kebede et al. (17)	2020	Addis Ababa City	Health facilities	Cross-sectional	238	127	53.36	97%	Low risk
Mohammed et al. (18)	2021	Harari	Jegel hospital	Cross-sectional	835	152	18.2	100%	Low risk

The extracted data were exported to Stata version 17 software from the Excel worksheet for further analysis. The funnel plot and Egger's regression test were used to assess publication bias through observation and *p*-value respectively. The pooled prevalence of second-trimester abortion and its associated factors was presented using a forest plot with a 95% confidence level. Cochran (Q test) and *I*^2^ test were used to assess random variation between primary studies (26). *I*^2^ was interpreted as no Heterogeneity, low heterogeneity, moderate heterogeneity, and high heterogeneity with an *I*^2^ value of 0%, 25%, 50%, and 75% respectively in this systematic review and meta-analysis (27). The derSimonian-Laird model was utilized in estimating the pooled prevalence of second-trimester induced abortion and its associated factors.

## Results

A total of 1,124 studies were retrieved through online databases including digital libraries of universities. Ninety studies were excluded due to duplicates, and 1,026 studies were excluded after a review of titles and abstracts giving 8 candidate studies for inclusion (Figure 1).

### Characteristics of included studies

Eight studies with a total study population of 3,659 were used to assess the pooled prevalence of second-trimester induced abortion and its associated factors in Ethiopia. Regional ways, the studies were conducted in Amhara, Oromia, South Ethiopia, Harari Regions, and Addis Ababa City Administration. The highest and the lowest prevalence of second-trimester induced abortion was reported in studies conducted in Addis Ababa City (17) and Harari Region (18) with respective magnitudes of 53.36% and 18.20% (Table 2).

### Meta-analysis

#### The prevalence of second-trimester-induced abortion

**Figure 2:**
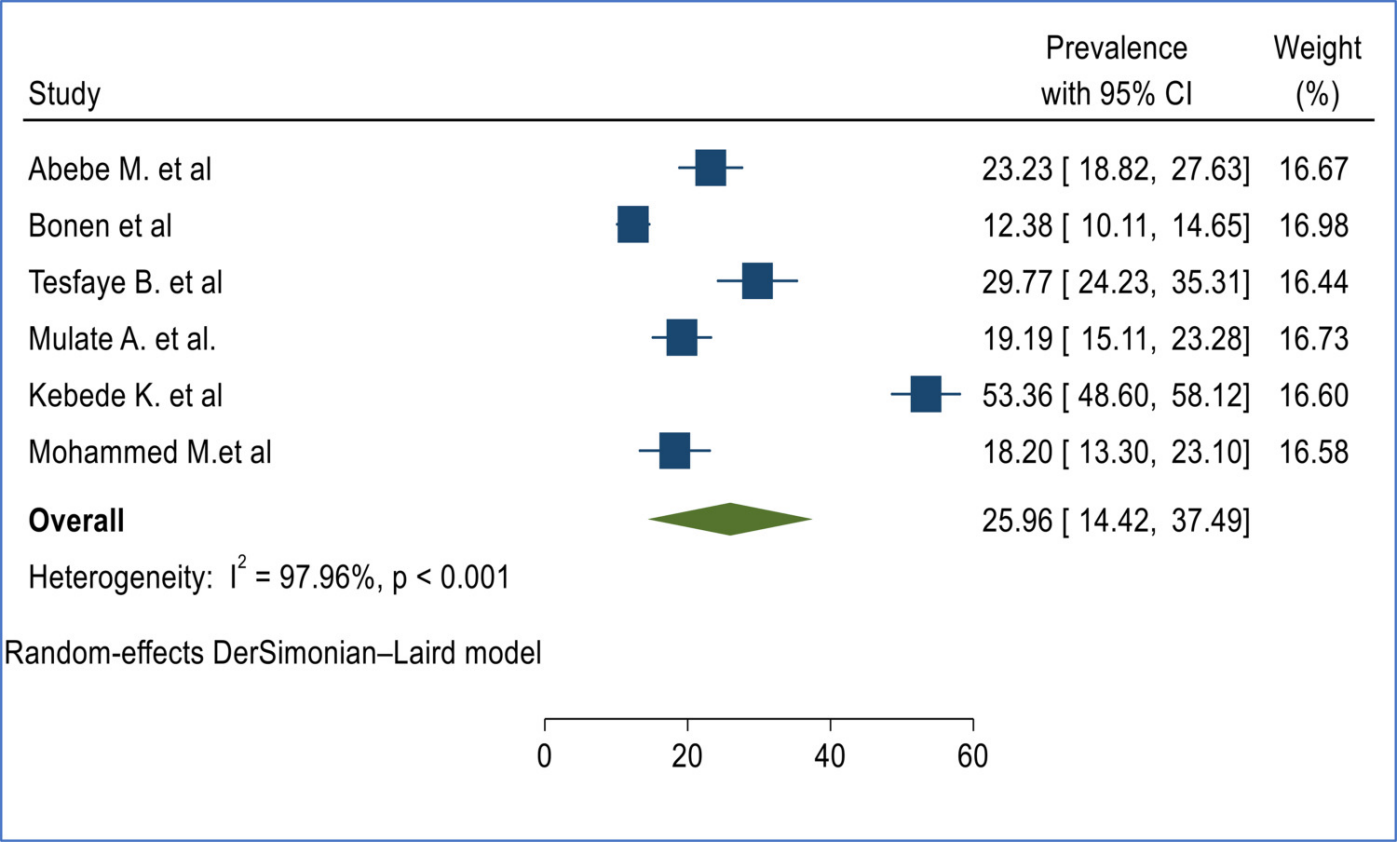
The pooled prevalence of second-trimester-induced abortion in Ethiopia.

The pooled prevalence of second-trimester induced abortion was 25.96% (95%, CI 14.42%, 37.49%) based on evidence extracted from six cross-sectional studies conducted from different Regions of Ethiopia (Figure 2).

### Sensitivity analysis

**Figure 3:**
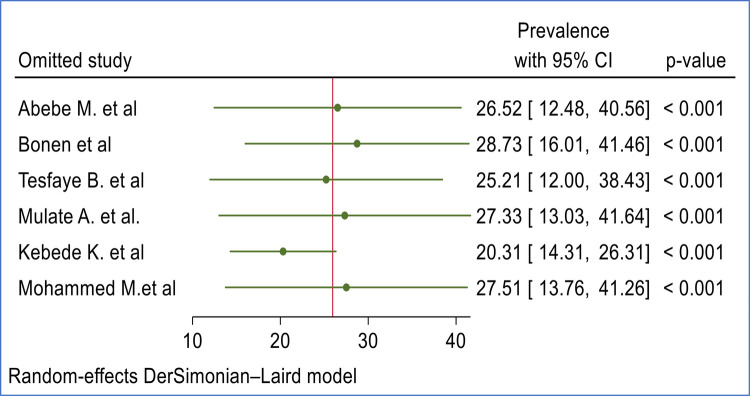
Sensitivity analysis of included studies to estimate the pooled prevalence second trimester induced abortion in Ethiopia.

Sensitivity analysis was performed to evaluate the influence of individual studies on the pooled prevalence of second-trimester induced abortion. The analysis indicated that there is no significant influence of individual studies on the pooled prevalence of second-trimester induced abortion (Figure 3).

### Heterogeneity

**Figure 4:**
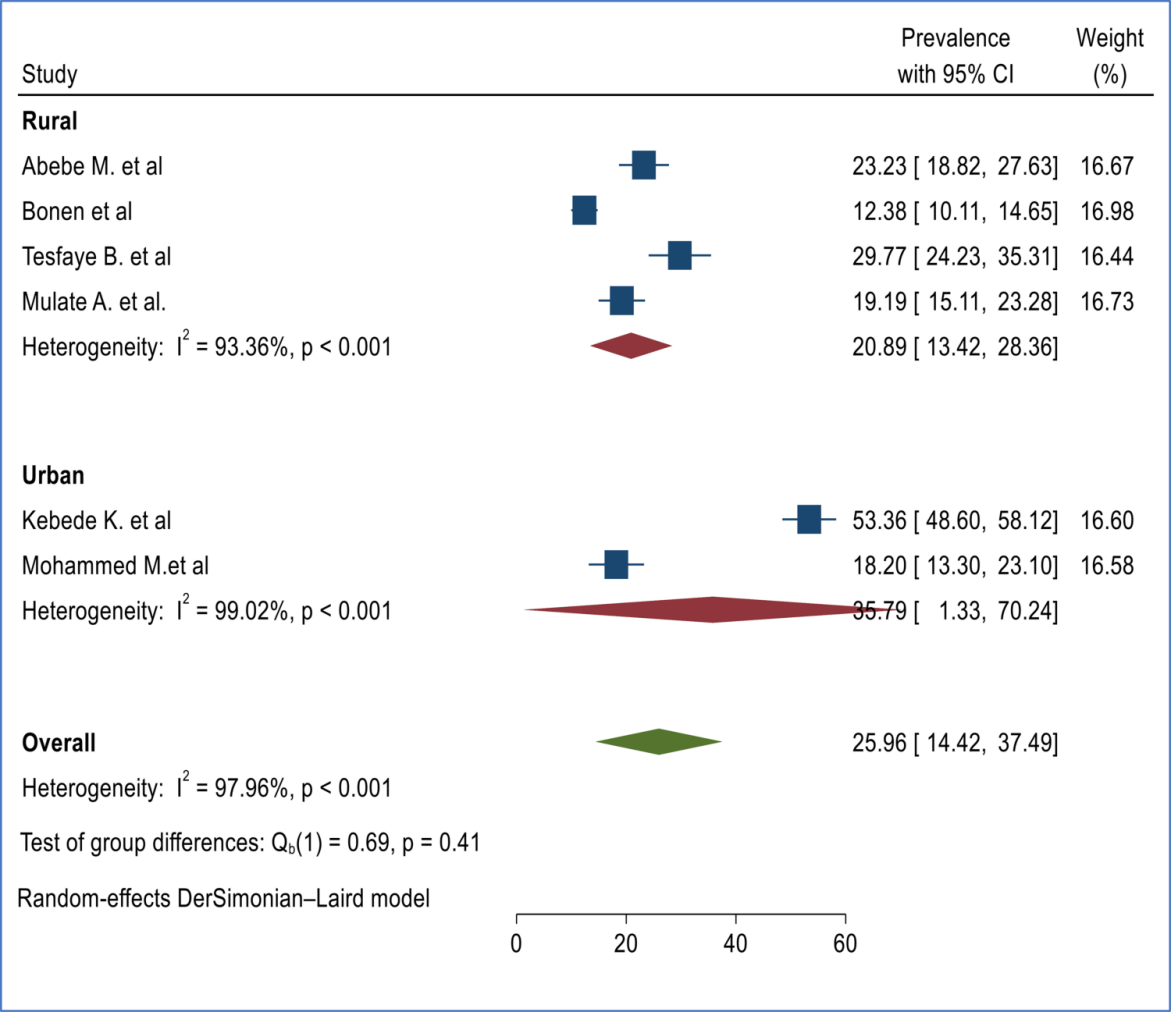
Subgroup analysis of burden of second trimester induced abortion in Ethiopia based on regions.

**Figure 5:**
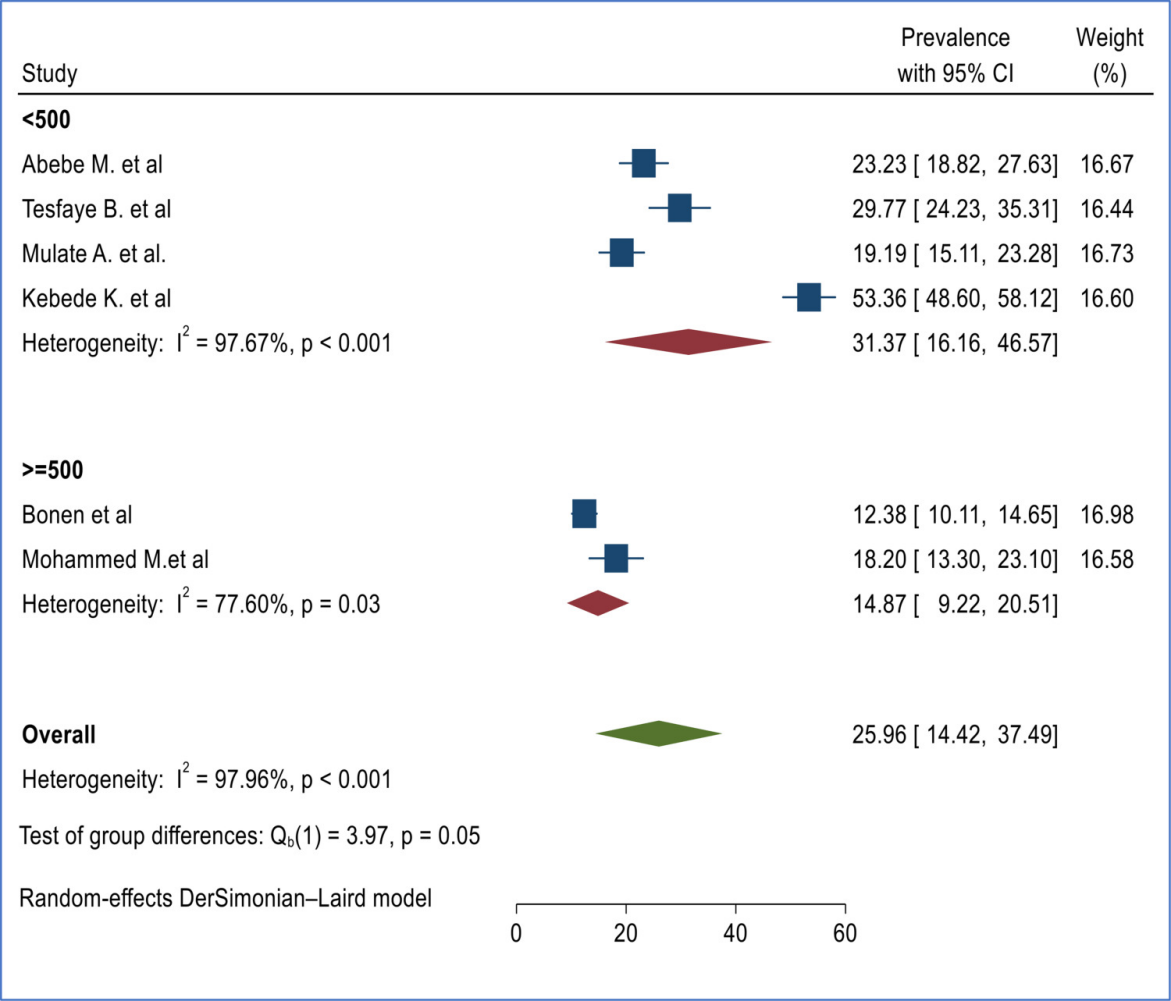
Subgroup analysis of burden of second-trimester abortion based on sample category in Ethiopia.

Subgroup analysis was conducted based on regions and sample size to assess the presence of heterogeneity between primary studies (Figures 4, 5).

### Subgroup analysis of the prevalence of induced second-trimester abortion in Ethiopia

Subgroup analysis was conducted based on study regions and sample size to identify the possible cause of heterogeneity. The subgroup analysis based on Regions (urban vs. rural) indicated that the prevalence of second-trimester abortion was found to be higher among urban centers 35.79% (95% CI 1.33%, 70.24%) (17, 18) compared with rural Regions 20.89% (95% CI 13.42%, 28.36%) (16, 19, 20, 25) though the difference was not statistically significant (Figure 4).

### Subgroup analysis based on sample size

Subgroup analysis was performed based on sample size by taking the mean sample size as a reference (≈500). The subgroup analysis based on sample size indicated that there is no significant difference among studies with a sample size of ≥500 (14.87% (95% CI 9.22%, 20.51%) (18, 25) and <500 (31.37% (16.16%, 46.57%) (16, 17, 19, 20) with significant heterogeneity between studies (Figure 5).

### Publication bias

**Figure 6:**
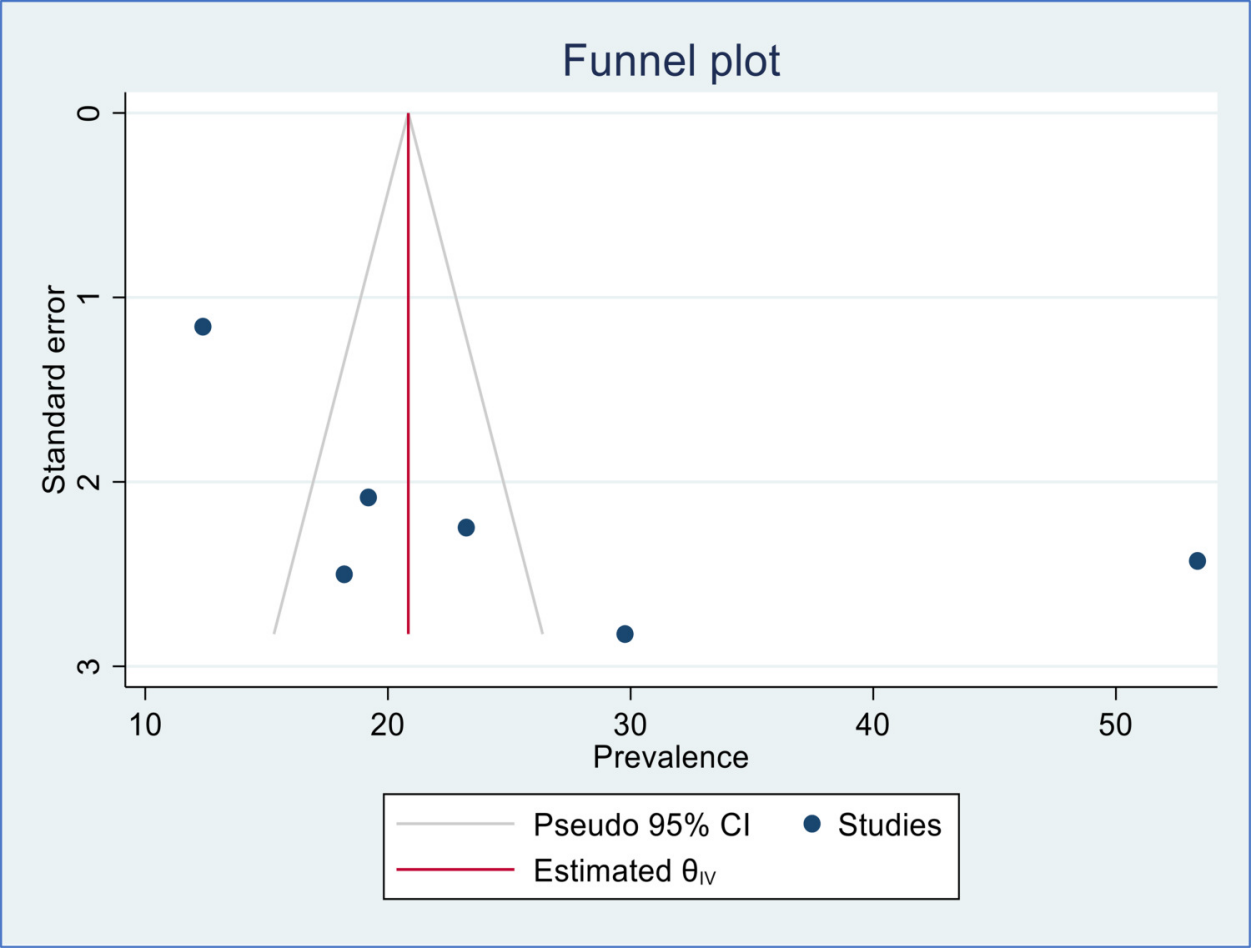
Funnel plot to assess publication bias of included studies in the meta-analysis of second-trimester induced abortion in Ethiopia.

We have used the funnel plot and Egger's regression asymmetry test to evaluate the presence of publication bias in this systematic review and meta-analysis. Hence there was no evidence of publication bias in this study based on evidence obtained from the funnel plot (Figure 6) and Egger's regression asymmetry test with *p*-value of 0.217.

### Factors associated with second trimester induced abortion in Ethiopia

**Figure 7:**
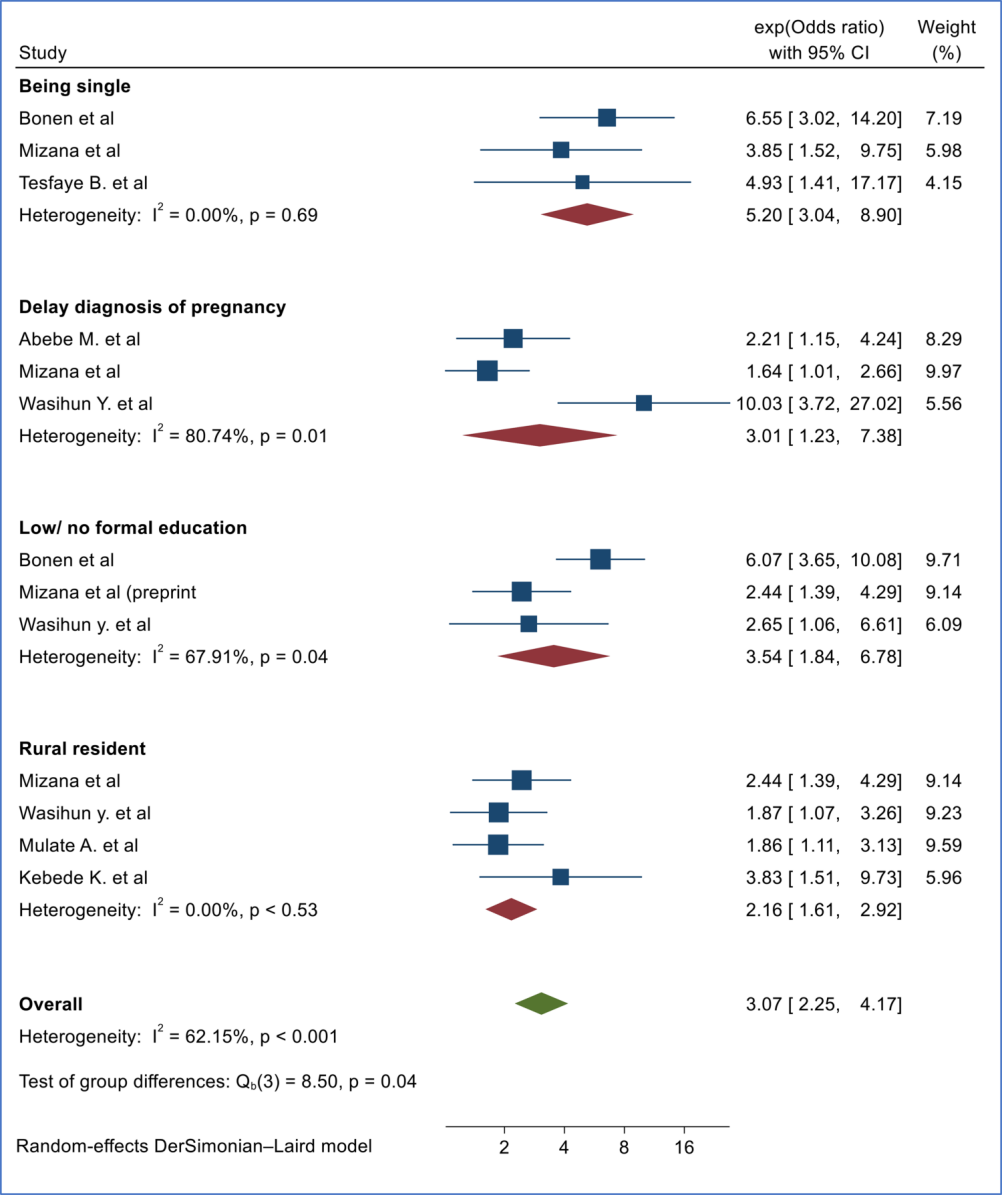
Forest plot of factors associated with second-trimester induced abortion in Ethiopia.

Based on the pooled evidence of primary studies conducted in different parts of Ethiopia, being single in marital status, delay in the diagnosis of pregnancy, having no formal/low education level, and being a rural resident were factors significantly associated with second-trimester-induced abortion in Ethiopia (Figure 7).

The marital status of the study participants was found to be significantly associated with induced second-trimester abortion based on the evidence compiled from three primary studies (15, 20, 25). The risk of experiencing second-trimester induced abortion is 5.2 times higher among single women compared to counterparts [(OR = 5.20, 95%, CI 3.04, 8.90), *I*^2^ = 0.00%, *p* = 0.69]. Similarly, women with delay in diagnosis of pregnancy had 3.01 times higher odds of experiencing induced second-trimester abortion compared to counterparts [(OR = 3.01, 95%, CI 1.23, 7.38), *I*^2^ = 80.74%, *p* = 0.01] based on the pooled finding of three primary studies (15, 16, 21) (Figure 7).

Based on the evidence from three primary studies each, women with no/low education level (15, 21, 25) [(OR = 3.54, 95%. CI 1.84, 6.78), *I*^2^ = 69.71, *p* = 0.04] and rural resident (15, 17, 19, 21) [(OR = 2.16, 95%, CI 1.61, 2.92), *I*^2^ = 0.00%, *p* = 0.53] were 3.35 and 2.16 times more likely to undergo induced second trimester induced abortion respectively compared to counterparts (Figure 7).

## Discussion

This study was conducted to assess the burden of second-trimester induced abortion and associated factors in Ethiopia. Based on the pooled evidence synthesized from six primary studies, the burden of second-trimester induced abortion was found to be 25.96% (95%, CI 14.42, 37.49%). The finding of our study is higher than the prevalence of second-trimester abortion in Zambia which reported a prevalence of 15% (28). In the same manner, the finding of our study was higher than the global second-trimester abortion prevalence of 10%–15% (29). The finding of this study highlights the necessity of reducing the incidence of second trimester abortions, either by offering family planning services to prevent abortions as a result of unwanted pregnancy or by ensuring that these services are accessible as early as possible during the first trimester (30, 31). The subgroup analysis indicated that there is no statistically significant difference in the prevalence of induced second-trimester abortion in Ethiopia based on study Regions and sample size category (16, 17, 19, 20, 25).

The risk of experiencing second-trimester induced abortion is 5.2 times higher among single women compared to their counterparts (OR = 5.20, 95%, CI 3.04, 8.90). The finding of our study is supported by a study conducted in the Volta Region of Ghana which reported a reduced risk of induced abortion among married women compared to unmarried women (32). A similar finding was also reported in a study conducted in Eastern Highlands Province, Papua New Guinea (33). The finding of this study suggested that unmarried women are more likely to be young, have an unmet need for family planning, and are more likely to have unwanted pregnancies than counterparts who end up with abortion (30, 34).

Similarly, women with delays in diagnosis of pregnancy had 3.01 times higher odds of experiencing induced second-trimester abortion compared to their counterparts (OR = 3.01, 95%, CI 1.23, 7.38). A similar finding was reported in previous studies unveiling delay in discovering pregnancy was higher among women who seek abortion services in the second trimester of pregnancy (35–37). Improving the sexual and reproductive health literacy of reproductive-age women as well as access to safe abortion services are relevant measures to be taken to reduce late visits to the health institutions for abortion services aside from preventing unwanted pregnancy through provision of family planning services (37–40).

The evidence obtained from three primary studies indicated that women with no/low education level were 3.54 times more likely to experience second-trimester induced abortion compared to their counterparts (OR = 3.54, 95%. CI 1.84, 6.78). However, the finding of our study is not supported by the finding of evidence obtained from evidence from the 2013 and 2019 Sierra Leone Demographic and Health Survey which reported an increased risk of induced abortion among educated women compared with uneducated counterparts (41). The difference might be attributed to the variation in the health system as well as the level of awareness of the people in the two nations.

Women who reported living in rural areas had 2.16 times higher odds of experiencing second-trimester abortion compared to urban counterparts (OR = 2.16, 95%, CI 1.61, 2.92). The finding of this study is supported by a study conducted in India which reported a threefold increased risk of second-trimester abortion among women who were forced to travel long times to health facilities. Improving access to health facilities that provide safe abortion services is imperative to reduce morbidity and mortality associated with second-trimester abortion (38).

### Strengths and limitations of the study

This systematic review has the following strengths; it synthesizes data from multiple studies conducted in all corners of the country, enhancing the reliability and generalizability of findings. The comprehensive approach allows for a deeper understanding of the prevalence and determinants of second trimester abortions, addressing critical gaps in existing literature. Moreover, the rigorous methodology employed strengthens the validity of the conclusions drawn, providing valuable insights for researches, policymakers, and healthcare providers.

The following limitations shall be considered while interpreting the findings of this systematic review and meta-analysis; we have used studies published in the English language only. On the other hand, a limited number of databases were utilized to search for potential studies. At the same time, the numbers of included studies in this systematic review and meta-analysis are limited which affects the generalizability of the finding.

## Conclusion

The prevalence of second-trimester abortion was found to be high in Ethiopia. Being single in marital status, delay in the diagnosis of pregnancy, having no formal/low education level, and being a rural resident were factors significantly associated with second-trimester induced abortion in Ethiopia. Enhancing the sexual and reproductive health literacy of reproductive-age women as well as improving access to safe abortion services are relevant measures to be taken to reduce late visits to the health institutions for abortion services. Future research should consider using larger sample sizes and a follow-up study design in this area.

## Data Availability

The original contributions presented in the study are included in the article/Supplementary Material, further inquiries can be directed to the corresponding author.
